# The Accuracy of Neutrophil-to-Lymphocyte Ratio and Abdominal Computed Tomography to Predict the Severity of Acute Cholecystitis

**DOI:** 10.7759/cureus.32243

**Published:** 2022-12-06

**Authors:** Gayathri Prakash, Mohammad Hasan

**Affiliations:** 1 Critical Care Medicine, Utkal Covid-19 Hospital, Keonjhar, IND; 2 Department of Internal Medicine, Jinnah Postgraduate Medical Centre, Karachi, PAK

**Keywords:** abdominal pain, radiology department, severe acute cholecystitis, ct scan, acute calculous cholecystitis

## Abstract

Background

In this study, we evaluated neutrophil-to-lymphocyte ratio (NLR) values and abdominal computed tomography (CT) scans in preoperative patients of acute cholecystitis (AC) and their significance in differentiating between severe and non-severe conditions. This study will help further in-depth investigation on both of these diagnostic modalities and timely assess severe AC to avoid the worst prognosis.

Methodology

This single-center, cross-sectional study was conducted at Government Villupuram Medical College from July 2021 to January 2022. We enrolled patients aged >18 years diagnosed with AC. The demographic variables and clinical features of the enrolled patients were collected. All enrolled patients were divided into two categories (severe or non-severe AC) based on the pathological and clinical findings. The data were collected and entered in SPSS Statistics version 26 (IBM Corp., Armonk, NY, USA). The variation between the severe and non-severe AC groups was compared using Student’s t-test to analyze continuous variables. The chi-square test was used to evaluate the association between the categorical variables. A p-value of <0.05 was considered significant.

Results

Among patients with severe AC, 29 (72.5%) were female, 29 (72.5%) were aged >50 years, 34 (85%) were alcoholics, and 26 (65%) were smokers. In patients with severe AC, the mean for NLR was 18.6500 ± 2.32655. On CT scans, 29 (72.5%) patients showed gallbladder distension, 31 (77.5%) showed increased pericholecystic fat stranding, and 18 (45%) showed pericholecystic fluid collection. CT scan findings and NLR values were significantly associated.

Conclusions

Gallbladder distension, increased pericholecystic fat stranding, and pericholecystic fluid collection on abdominal CT scan along with raised NLR are significant findings associated with assessing the severity of AC. Therefore, both testing modalities (CT scan and NLR) should be utilized together in hospitals to achieve better outcomes for AC and avoid complications.

## Introduction

Cholecystitis is one of the most common and frequent presenting complaints in gastroenterology [1]. The acute condition usually occurs due to the obstruction of gallstones in the cystic duct. Usually, a gallstone blocking cystic duct opening leads to intense contractions of the gallbladder causing colic pain. If gallstones remain in the duct they can cause further contractions that lead to edema of the gallbladder walls. This edema can initiate damage to the epithelial layer which increases the secretion of enzymes such as phospholipase which cause inflammation. This acute inflammation can cause the gallstone to pass or seize the gall muscles [2]. In this situation, the possibility of bacterial infection increases which leads to further complications such as sepsis, gangrene of the gall bladder wall, and perforation [3]. Overall, 25% of patients may present with acute acalculous cholecystitis. Most patients may present with previous history such as pain in the epigastric region radiating to the back and referred to the shoulder region, nausea, and complaints of heartburn and indigestion [4]. Presenting symptoms may also include positive Murphy’s sign, fever, and increased white blood cell (WBC) count [5].

For prognosis of inflammatory diseases, modified Glasgow prognostic score, neutrophil-to-lymphocyte ratio (NLR), platelet-to-lymphocyte ratio, and prognostic nutritional index are utilized. Among these, NLR is preferred because it is simple and inexpensive. It can be calculated by a complete blood count test [6]. The NLR is measured by dividing the total count of neutrophils by the total count of lymphocytes considered to be a biomarker for inflammation [7]. Raised NLR levels are also seen in many acute inflammatory conditions such as appendicitis and severe traumatic and septic shock. In acute cholecystitis (AC), as it is also an inflammatory condition, NLR is used as a biomarker [8]. Previously, WBC count and C-reactive protein (CRP) level was used for diagnosis; however, because these results can be normal in AC patients, for more accuracy in inflammatory indication, NLR is used [8].

Radiological studies to diagnose AC include ultrasound of the abdomen, computed tomography (CT), magnetic resonance imaging (MRI), and hepatobiliary iminodiacetic acid (HIDA) scan [2]. Abdominal CT is largely used in emergency cases of abdominal pain for unknown reason [9].

The standard treatment for AC is cholecystectomy. Medications are used to reduce inflammation and eliminate bacterial infections [10]. Laparoscopic cholecystectomy is now commonly used compared to open surgery because of less scarring, reduced postoperative pain, shorter duration of hospitalization, and fast recovery [11].

In this study, we studied the NLR and abdominal CT scan in preoperative AC patients and their significance in differentiating between severe and non-severe conditions. We also studied the best and standard imaging tests used to diagnose the condition. This study will help further in-depth research regarding these diagnostic modalities and timely assess severe AC to avoid the worst prognosis.

## Materials and methods

This single-center, cross-sectional study was conducted at Government Villupuram Medical College from July 2021 to January 2022. We enrolled patients aged >18 years diagnosed with AC by physicians based on clinical signs and symptoms that include positive Murphy’s sign, right upper quadrant (RUQ) pain, diffuse abdominal pain, epigastric pain, fever (≥100°F), elevated CRP (3 mg/dL or more), elevated WBC (>11.0 × 10^9^/L), and CT scan imaging (gallbladder stone, gallbladder wall thickening, perihepatic hyperattenuation, pericholecystic fluid collection, gallbladder distension, and increased density of pericholecystic fat stranding). Patients who refused to give consent or did not undergo the CT examination and were admitted for elective cholecystectomy, those whose diagnosis was not clear, those who had consumed antibiotics within the last two weeks before visiting the hospital, or those who were immunocompromised (e.g., human immunodeficiency virus, malignancy) were excluded from the study. AC was diagnosed by internal medicine physicians or biliary surgeons by evaluating the clinical signs, investigation findings, and CT scan findings. Using the Raosoft sample size calculator with an error margin of 5%, a confidence interval of 95%, and an expected population size of 156 [12], the sample size was calculated to be 112 patients.

Ethical approval was sought from the Institutional Review Board of Government Villupuram Medical College (reference number: GVMC/IRB/2021/0022). Informed consent was taken from all patients. The demographic variables and clinical features of enrolled patients were collected, including their age, final diagnosis, cholecystectomy operation, duration of the hospital stay, and intensive care unit (ICU) admission. The clinical examination findings were also collected which included fever, RUQ pain, duration of symptoms, epigastric pain, Murphy’s sign, early blood examination (neutrophil count, NLR, WBC count, erythrocyte sedimentation rate (ESR), CRP, aspartate aminotransferase (AST), alanine transaminase (ALT), prothrombin time (PT), international normalized ratio (INR)) within the initial hours of the hospital admission. An abdominal CT (contrast or non-contrast) was also obtained within six hours of the hospital admission. As part of the hospital protocol, it was assessed by the doctor whether the patient needed a CT scan with or without contrast depending on the patient’s condition and medical history related to kidney issues. The CT scan findings that were collected included gallbladder stone, gallbladder wall thickening, perihepatic hyperattenuation, pericholecystic fluid collection, gallbladder distension, and increased density of pericholecystic fat stranding.

All enrolled patients were divided into two categories (severe or non-severe AC) based on the pathological and clinical findings. Severe AC was defined as the presence of pathologic gangrenous, necrotizing, suppurative, and perforated cholecystitis. In cases where the patients were not undergoing cholecystectomy, they were categorized based on the 2007 Tokyo Guidelines (TG07) [13] for severe AC (Grade III).

Patients with AC were divided into three categories (Grades I, II, and III) using the TG07 and the comparison of the severe and non-severe AC groups. The division into the different grades was as follows [14]: Grade I included cases with no organ dysfunction and only mild inflammatory changes in the GB; Grade II included patients who had one of the following conditions: WBC count >18,000/mm^3^, pain duration >72 hours, a palpable tender mass in the RUQ, pain duration >72 hours, or marked local inflammation; Grade III included patients who had AC accompanied by organ/system dysfunction. The diagnosis of severe Grade III AC was made when one or more symptoms of the organ or functional failure were noted.

The data were collected and entered in SPSS Statistics version 26 (IBM Corp., Armonk, NY, USA). The variation between the severe and non-severe AC groups was compared using Student’s t-test to analyze continuous variables. The chi-square test was used to evaluate the association between the categorical variables. A p-value of <0.05 was considered significant.

## Results

Table 1 shows the demographic variables of patients suffering from severe and non-severe AC. Among the patients with severe AC, 29 (72.5%) were female, 29 (72.5%) were aged >50 years, 34 (85%) were alcoholics, and 26 (65%) were smokers. None of these variables were significantly associated with the severity of AC.

**Table 1:** Demographic variables of patients suffering from severe and non-severe acute cholecystitis. BMI = body mass index

Variables	Categories	Non-severe (n = 72)	Severe (n = 40)	P-value
Gender	Male	26 (36.1%)	11 (27.5%)	0.353
	Female	46 (63.9%)	29 (72.5%)	
Age	18–30	5 (6.9%)	2 (5%)	0.862
	31–50	18 (25%)	9 (22.5%)	
	>50	49 (68%)	29 (72.5%)	
BMI	>25	55 (76.4%)	30 (75%)	0.869
	<25	17 (23.6%)	10 (25%)	
Alcohol	Yes	54 (75%)	34 (85%)	0.216
	No	18 (25%)	6 (15%)	
Smoking	Yes	34 (47.2%)	26 (65%)	0.07
	No	38 (52.7%)	14 (35%)	

Table 2 shows the clinical findings and features of patients having severe and non-severe AC. Among the patients with severe AC, 28 (70%) were having symptoms for >24 hours, 24 (60%) had a fever, 26 (65%) had undergone cholecystectomy, 25 (62.5%) patients were reported to be severe on TG07 severity grading score, 34 (85%) were admitted in the ICU, and eight (20%) died. All of these variables were significant with a p-value of <0.05.

**Table 2:** Clinical findings and features of patients having severe and non-severe acute cholecystitis. RUQ = right upper quadrant; TG07 = 2007 Tokyo Guidelines; ICU = intensive care unit *: P-value <0.05

Clinical findings	Categories	Non-severe (n = 72)	Severe (n = 40)	P-value
Duration of symptoms (hours)	>24	35 (48.6%)	28 (70%)	0.028*
	<24	37 (51.3%)	12 (30%)	
Fever	Yes	19 (26.4%)	24 (60%)	0.000*
	No	53 (73.6%)	16 (40%)	
RUQ pain	Yes	59 (81.9%)	35 (87.5%)	0.443
	No	13 (18.1%)	5 (12.5%)	
Epigastric pain	Yes	62 (86.1%)	33 (82.5%)	0.297
	No	10 (13.9%)	9 (22.5%)	
Murphy’s sign	Yes	28 (38.9%)	13 (32.5%)	0.501
	No	44 (61.1%)	27 (67.5%)	
Diffuse abdominal pain	Yes	18 (25%)	11 (27.5%)	0.772
	No	54 (75%)	29 (72.5%)	
Cholecystectomy	Yes	31 (43.1%)	26 (65%)	0.026*
	No	41 (56.9%)	14 (35%)	
TG07 severity grading	Mild	40 (55.5%)	2 (5%)	<0.001*
	Moderate	30 (41.6%)	13 (32.5%)	
	Severe	2 (2.8%)	25 (62.5%)	
ICU admission	Yes	1 (1.4%)	34 (85%)	<0.001*
Deaths	Yes	2 (2.8%)	8 (20%)	0.002*

Table 3 shows the values of the biomarkers and the CT scan findings. Among patients with severe AC, the mean neutrophil count was 14.4 ± 4.03, the mean NLR was 18.65 ± 2.32, the mean platelets were 233.4 ± 43.8, the mean ALT was 46.75 ± 19.28, the mean PT/INR was 1.24 ± 0.37, the mean ESR was 61.27 ± 13.52, and the mean CRP was 116.1 ± 30.6. In the CT scan findings, patients with severe AC revealed 29 (72.5%) had gallbladder distension, 31 (77.5%) had increased pericholecystic fat stranding, and 18 (45%) had pericholecystic fluid collection.

**Table 3:** Values of the biomarkers and computed tomography scan findings. WBC = white blood cell; NLR = neutrophil-to-lymphocyte ratio; AST = aspartate transaminase; ALT = alanine transaminase; PT = prothrombin time; INR = international normalized ratio; ESR = erythrocyte sedimentation rate; CRP = C-reactive protein *: P-value < 0.05

Laboratory finding	Non-severe (n = 72)	Severe (n = 40)	P-value
WBC count (×10^9^/L)	8.01 ± 2.59736	16.3000 ± 2.43057	0.927
Neutrophil count (×10^9^/L)	8.86 ± 2.99439	14.4000 ± 4.03065	0.001*
NLR	8.20 ± 2.22620	18.6500 ± 2.32655	0.044*
Platelet	217.6 ± 23.77628	233.4000 ± 43.84577	0.021*
AST (U/L)	66.04 ± 32.81508	51.2250 ± 32.92298	0.169
ALT (U/L)	59.84 ± 12.92084	46.7500 ± 19.28165	0.000*
PT INR	1.68 ± 0.72823	1.2475 ± 0.37071	0.000*
ESR (mm/hour)	31.41 ± 7.49413	61.2750 ± 13.52299	0.000*
CRP (mg/L)	36.72 ± 8.06177	116.1500 ± 30.61050	0.000*
Gallbladder stone, n (%)	33 (45.8%)	24 (60.0)	0.1507
Gallbladder distension, n (%)	32 (44.4%)	29 (72.5%)	0.0042*
Gallbladder wall thickening, n (%)	31 (43%)	16 (40%)	0.753
Perihepatic hyperattenuation, n (%)	10 (13.9%)	7 (17.5%)	0.609
Increased pericholecystic fat stranding, n (%)	16 (22.2%)	31 (77.5%)	<0.001*
Pericholecystic fluid collection, n (%)	4 (5.5%)	18 (45%)	<0.001*

## Discussion

We conducted this study to evaluate which modality, namely, NLR or CT scan, is more appropriate in predicting the clinical severity of AC among all patients with AC. In our study, the majority of the patients were females (66.9%), which is consistent with the high female-to-male ratio of AC reported in another study [12]. In our study, among patients with severe AC, 70% had a symptom duration of >24 hours, 60% had a fever, and 65% had undergone cholecystectomy. These findings were consistent with the findings of the study conducted by Woo et al. [12].

The surgery-related trauma results in a metabolic and inflammatory response. The body’s reaction to tissue damage varies depending on the severity of the surgical trauma [14]. Because more complications are associated with severe AC compared to simple AC, early detection of severe AC is crucial to avoid perioperative metabolic and inflammatory complications and longer hospital stays. Usually, for a diagnostic procedure, CT is not always sensitive enough to indicate the presence of severe AC [15]. The literature suggests that certain biomedical markers can be potentially useful predictors of the severity of AC.

Hematological and biochemical factors are good predictors of severe inflammation. These markers include WBC and CRP. Although WBC count is a cost-effective and good indicator of inflammation, it lacks the ability the assess the severity of AC [16]. NLR is a combination of two markers, with neutrophils as an active non-inflammatory mediator resulting in the first-line defense mechanism while lymphocytes as the regulatory or protective molecule of the inflammation [17,18]. NLR is an inexpensive and convenient to assess marker which can be measured during routinely determined cell blood counts. According to one of the studies, NLR is more accurate in predicting poor postoperative outcomes compared to measuring WBC subcategories individually [19]. In our study, NLR was found to be significantly associated with predicting the severity of AC along with the significant findings of CT scans, including gallbladder distension, increased pericholecystic fat stranding, and pericholecystic fluid collection.

The strength of our study was that it assessed the accuracy of CT scans and NLR in predicting the severity of AC and compared the findings.

The major limitation of our study was that the data were collected from a single institute due to which additional variables and demographic factors were not included and a smaller sample size was achieved. Another limitation was that the standard imaging technique was not utilized. If all patients were administered intravenous contrast CT scans, it would have been better in detecting the inflammation and severity. Another limitation of our study was that we did not include whether the gallbladder was gangrenous, necrotizing, or suppurative for patients who were not suitable for cholecystectomy.

## Conclusions

Gallbladder distension, increased pericholecystic fat stranding, and pericholecystic fluid collection on the abdominal CT scan along with raised NLR are significant findings associated with assessing the severity of AC. Therefore, both testing modalities (CT and NLR) should be utilized together in hospitals to achieve better outcomes for AC and avoid complications.

## References

[1] Russo MW, Wei JT, Thiny MT (2004). Digestive and liver diseases statistics, 2004. Gastroenterology.

[2] Sharp KW (1988). Acute cholecystitis. Surg Clin North Am.

[3] Fukunaga FH (1973). Gallbladder bacteriology, histology, and gallstones. Study of unselected cholecystectomy specimens in Honolulu. Arch Surg.

[4] Gagic N, Frey CF (1975). The results of cholecystostomy for the treatment of acute cholecystitis. Surg Gynecol Obstet.

[5] Micić D, Stanković S, Lalić N, Đukić V, Polovina S (2018). Prognostic value of preoperative neutrophil-to-lymphocyte ratio for prediction of severe cholecystitis. J Med Biochem.

[6] Tamhane UU, Aneja S, Montgomery D, Rogers EK, Eagle KA, Gurm HS (2008). Association between admission neutrophil to lymphocyte ratio and outcomes in patients with acute coronary syndrome. Am J Cardiol.

[7] Lee SK, Lee SC, Park JW, Kim SJ (2014). The utility of the preoperative neutrophil-to-lymphocyte ratio in predicting severe cholecystitis: a retrospective cohort study. BMC Surg.

[8] Beliaev AM, Angelo N, Booth M, Bergin C (2017). Evaluation of neutrophil-to-lymphocyte ratio as a potential biomarker for acute cholecystitis. J Surg Res.

[9] Fagenholz PJ, Fuentes E, Kaafarani H (2015). Computed tomography is more sensitive than ultrasound for the diagnosis of acute cholecystitis. Surg Infect (Larchmt).

[10] van der Linden W, Sunzel H (1970). Early versus delayed operation for acute cholecystitis. A controlled clinical trial. Am J Surg.

[11] Vemulapalli P, Agaba EA, Camacho D (2011). Single incision laparoscopic cholecystectomy: a single center experience. Int J Surg.

[12] Woo SH, Lee WJ, Seol SH, Kim DH, Choi SP (2018). The accuracies of abdominal computed tomography and the neutrophil-to-lymphocyte ratio used to predict the development of clinically severe acute cholecystitis in elderly patients visiting an emergency department. Niger J Clin Pract.

[13] Hirota M, Takada T, Kawarada Y (2007). Diagnostic criteria and severity assessment of acute cholecystitis: Tokyo Guidelines. J Hepatobiliary Pancreat Surg.

[14] Yuksel M, Ates I, Kaplan M (2017). Is oxidative stress associated with activation and pathogenesis of inflammatory bowel disease?. J Med Biochem.

[15] Parker LJ, Vukov LF, Wollan PC (1997). Emergency department evaluation of geriatric patients with acute cholecystitis. Acad Emerg Med.

[16] Ahmed SE, Rehman S, Edilbe M, Jonker L, Ruben C (2017). Can neutrophil-lymphocyte ratio predict operators’ difficulty in early cholecystectomies; a retrospective cohort study. Ann Emerg Surg.

[17] Bhutta H, Agha R, Wong J, Tang TY, Wilson YG, Walsh SR (2011). Neutrophil-lymphocyte ratio predicts medium-term survival following elective major vascular surgery: a cross-sectional study. Vasc Endovascular Surg.

[18] Kang HS, Cha YS, Park KH, Hwang SO (2017). Delta neutrophil index as a promising prognostic marker of emergent surgical intervention for acute diverticulitis in the emergency department. PLoS One.

[19] Abdel-Razik A, Mousa N, Shabana W (2016). A novel model using mean platelet volume and neutrophil to lymphocyte ratio as a marker of nonalcoholic steatohepatitis in NAFLD patients: multicentric study. Eur J Gastroenterol Hepatol.

